# Clinical outcomes and toxicity using Stereotactic Body Radiotherapy (SBRT) for advanced cholangiocarcinoma

**DOI:** 10.1186/1748-717X-7-67

**Published:** 2012-05-03

**Authors:** Brandon M Barney, Kenneth R Olivier, Robert C Miller, Michael G Haddock

**Affiliations:** 1 Department of Radiation Oncology, Mayo Clinic, Rochester, MN, USA; 2 Mayo Clinic, 200 First Street SW, Rochester, MN, 55905, USA

**Keywords:** Stereotactic body radiotherapy, Stereotactic radiosurgery, Cholangiocarcinoma, Locally advanced

## Abstract

**Background:**

To report single-institutional clinical outcomes and toxicity with SBRT for cholangiocarcinoma.

**Methods:**

From March 2009 to July 2011, 10 patients with 12 unresectable primary (n = 6) or recurrent (n = 6) cholangiocarcinoma lesions underwent abdominal SBRT. Sites treated included liver (n = 10), abdominal lymph nodes (n = 1), and adrenal gland (n = 1). SBRT was delivered in three (n = 2) or five (n = 10) consecutive daily fractions over one week. The median prescription dose was 55 Gy (range, 45–60). Treatment response was graded by RECIST v.1.1, and toxicities were scored by CTCAE v.4.0. Data was analyzed using the Kaplan-Meier method to determine rates of local control (LC), freedom from distant progression (FFDM) and overall survival (OS).

**Results:**

The median follow-up was 14 months (range, 2–26 months). LC, defined as freedom from progression within the SBRT field, was 100%, but four patients treated to intrahepatic sites experienced progression elsewhere in the liver. Estimates for FFDM at 6 and 12 months were 73% and 31%, respectively. Sites of disease relapse included liver (n = 3), liver and lymph nodes (n = 1), liver and lungs (n = 1), lymph nodes (n = 1), and mesentery (n = 1). OS estimates for the cohort at 6 and 12 months were 83% and 73%, respectively. The most common Grade ≥2 early toxicities were Grade 2 nausea and vomiting (n = 5) and gastrointestinal pain (n = 2). Late ≥2 toxicities included Grade 2 gastrointestinal pain (n = 3), Grade 3 biliary stenosis (n = 1), and Grade 5 liver failure (n = 1).

**Conclusions:**

SBRT shows promise as an effective local therapy for properly-selected patients with cholangiocarcinoma. Further follow-up is needed to better quantify the risk of late complications associated with SBRT.

## Background

Cholangiocarcinoma is a rare, locally-aggressive malignancy of the biliary tree that is traditionally classified as either intra- or extra-hepatic, based on its location within the bile ducts. Currently, the only known curative therapy for cholangiocarcinoma is surgical resection [1,2]. Unfortunately, the majority of patients present with unresectable disease, and even when all disease is amenable to resection, local recurrence is common [1,3]. Non-surgical options for patients with unresectable or recurrent disease include combinations of chemotherapy, external beam radiotherapy (EBRT), transarterial chemoembolization (TACE), radiofrequency ablation (RFA), and/or photodynamic therapy (PDT) [4-8], although EBRT is the most common local therapy utilized for patients without metastatic disease. Yet even with this diverse array of treatment modalities, the median survival for locally-advanced or recurrent cholangiocarcinoma is approximately 9 months, and 5-year overall survival is less than 5% [9].

While the majority of patients eventually progress within the high-dose region when treating with standard-fractionation EBRT [5], most contemporary series have demonstrated a clinical dose–response relationship [5,10,11]. This has resulted in a renewed interest in dose-escalation as a means of improving disease control and survival. One way in which dose escalation may be achieved is through the use of stereotactic body radiotherapy (SBRT), which involves the delivery of very high doses of highly conformal radiotherapy in five or fewer fractions. Some early reports of SBRT for unresectable cholangiocarcinoma have shown equivalent or improved rates of local control compared to EBRT, albeit with increased toxicity [12-16]. Since 2008, we have enrolled patients with unresectable or recurrent cholangiocarcinomas treated with SBRT in a prospectively-maintained institutional database. In this study, we present the early clinical outcomes and toxicity associated with SBRT for unresectable or recurrent cholangiocarcinoma.

## Methods

### Patients

This study was approved by the Mayo Clinic Institutional Review Board (IRB) and appropriate informed consent was obtained prior to treatment. All clinical data was obtained from the prospective institutional SBRT database. This database is maintained through continued review by the institutional IRB, and all patients give informed consent prior to being included therein. No patient in this study was treated on protocol. All prospectively collected data was confirmed and analyzed retrospectively for the purposes of this review. All patients had a biopsy-proven cancer diagnosis and had undergone complete staging evaluation with history, physical exam, and full-body imaging with either computed or positron emission tomography. Patients with metastatic cholangiocarcinoma were considered for SBRT as long as all active disease was amenable to some form of local therapy, or if the patient had symptomatic disease progression. Appropriate imaging of the lesion(s) in question was required, which typically consisted of magnetic resonance imaging (MRI) or triple-phase contrasted computed tomography (CT) for intrahepatic lesions and regular CT, with or without contrast, for non-liver lesions. The decision to undertake a course of SBRT was made in a multidisciplinary fashion--patients were presented with all treatment options prior to making the choice to proceed with SBRT. No other specific criteria were used to select patients with cholangiocarcinoma for SBRT.

### SBRT technique

Patients were immobilized using the Body-Fix whole-body immobilization system (Medical Intelligence, Schwabmünchen, Germany). Axial CT planning images were obtained on a General Electric (GE) Light Speed RT 16-slice CT simulator (GE HealthCare, Fairfield, CT) with four-dimensional (4D-CT) respiratory monitoring via an infrared reflector placed on the patient’s chest (Varian RPM, Varian, Palo Alto, CA). The patient’s respiratory pattern and CT data were linked at the time of simulation and transferred to an Advantage virtual simulation station (GE HealthCare) for tumor motion evaluation and isocenter placement. Techniques such as respiratory gating or abdominal compression were reserved for patients whose 4D-CT images demonstrated >5 mm of tumor respiratory motion.

Normal tissue and tumor segmentation was performed on the Advantage workstation. In all cases, planning imaging was fused with diagnostic imaging to aid in tumor and normal tissue delineation. Gross tumor volume (GTV) was considered to be identical to clinical target volume (CTV). CTV was modified to create an internal target volume (ITV), accounting for the movement of the tumor in three dimensions using 4D-CT images. This ITV was not generated solely by contouring on maximum intensity projection (MIP) images, but rather by contouring on the binned image sets chosen from specific time points during the patient’s breathing cycle. ITV contours were then reviewed by playing back the entire 4D image loop to ensure that the entire tumor volume was contained within the ITV contour at all phases of the breathing cycle. The planning target volume (PTV) was typically created using a uniform 5 mm expansion of the ITV in all dimensions. Normal tissues at risk, including skin, spinal cord, stomach, small and large bowel, duodenum, kidneys and liver were segmented. In some patients with liver tumors not readily visible on a non-contrasted CT scan, 2 to 6 small fiducials were placed percutaneously using ultrasound guidance around the tumor borders under local anesthesia by an interventional radiologist. Image-guided radiotherapy was accomplished with either cone-beam computed tomography (CBCT) or stereoscopic kV imaging of fiducials. Daily imaging was acquired prior to each treatment, a match was performed, and the shift was applied. Any shift greater than 0.3 cm required a second image acquisition to verify position.

Patients were treated with either 3 or 5 fractions on consecutive days, with efforts made to complete therapy within a single week. The dose prescribed and number of fractions delivered was at physician discretion and was based upon target proximity to critical structures. Heterogeneity corrections were used in all cases. Both IMRT and 3D-conformal SBRT treatment plans were designed using Eclipse (Varian) treatment planning software. Often when IMRT was used, a heterogenous dose distribution was achieved by prescribing a higher dose to GTV relative to the PTV, creating a cloud of increased dose around the gross disease while facilitating the attainment of constraints on normal tissues in the immediate vicinity. Dose constraints used on organs at risk were somewhat dependent on SBRT fractionation, but can be generalized as follows: for bowel structures (stomach, duodenum, and intestine), a maximum point dose of 32 Gy and a 10 cc constraint of 20 Gy; for liver, at least 700 cc of normal liver to receive less than 21 Gy; for kidney, at least 200 cc of each kidney to receive less than 17.5 Gy; and for spinal cord, a maximum dose of 20 Gy.

### Toxicity and follow-up

Follow-up consisted of imaging of the treated area, a clinical evaluation, and appropriate laboratory testing 2–3 months after completing SBRT. Follow-up thereafter occurred at 3–6 month intervals with imaging studies. Tumor treatment response was scored by a radiation oncologist using the Response Evaluation Criteria in Solid Tumors (RECIST) v1.1 [17], and toxicity was scored using the National Cancer Institute (NCI) Common Terminology Criteria for Adverse Events (CTCAE) v3.0.

### Statistics

The Kaplan-Meier (KM) method was used to define rates of overall survival (OS), local control (LC), and freedom from distant progression (FFDM). Distant failure was defined as the development of new metastases or progression of untreated metastases. Survival and control times were calculated from the end of SBRT.

## Results

### Patient and treatment characteristics

Baseline patient and disease characteristics for the 10 patients are available in Table 1. The median patient age was 61.6 years (range, 51.4 to 87.0 years). The initial primary cholangiocarcinoma location was intrahepatic (n = 6) or extrahepatic (n = 4). Three of the four extrahepatic tumors were located in the perihilar region (Klatskin tumor). Six of the 12 treated lesions (50%) consisted of unresectable primary sites, and the other 6 lesions (50%) were recurrent after initial surgery, either in the liver (n = 5) or adrenal gland (n = 1). All primary site lesions were biopsy-proven prior to SBRT, and all patients with recurrent disease had, at minimal, imaging evidence of disease recurrence on PET-CT and/or MRI.

**Table 1:** Baseline patient and tumor characteristics

**Characteristic**	**Value**
Patients (n)	10
Lesions (n)	12
Age (y)	
Median	61.6
Range	51.4-87.0
Gender (n)	
Male	6
Female	4
Initial tumor location	
Intrahepatic	6
Extrahepatic	4

The median interval from the time of initial diagnosis to SBRT was 2.3 years (range, 0–5 years). For patients who had previously undergone surgical resection, the time from surgery to disease recurrence treated with SBRT was 3.7 years (range, 2–5 years). A total of 4 patients had received systemic therapy at some point prior to SBRT, and 4 patients also received systemic therapy after completion of SBRT. One patient treated for a recurrent liver metastasis had previously received adjuvant standard fractionation EBRT to the primary site and regional lymph nodes after resection of an extrahepatic cholangiocarcinoma. No other patient had received previous EBRT.

Treatment details, including site treated, prescribed dose, and PTV volume and dose information is described in Table 2. The median prescribed dose was 55 Gy in 5 fractions (range, 45–60 Gy in 3–5 fractions). All but 2 patients were treated in 5 fractions with total doses of 45, 50 or 60 Gy. The choice of dose was based on tumor volume and proximity of dose limiting structures. The median PTV volume was 79.1 cm^3^ (range, 16.0-412.4 cm^3^).

**Table 2:** Treatment characteristics

**Patient**	**Age**	**Site treated**	**Prescribed dose (Gy)**	**Fractions (n)**	**PTV volume (cc)**	**PTV maximum dose (Gy)**	**PTV minimum dose (Gy)**	**PTV mean dose (Gy)**
1	56	Liver	50	5	30.5	59.8	35.8	55.3
	57	Liver	45	5	16.0	49.4	36.5	46.4
	57	Lymph node	45	5	30.4	49.1	34.9	45.7
2	59	Liver	50	5	412.4	56.8	35.0	53.4
3	69	Liver	60	3	67.0	68.9	52.9	65.4
4	82	Liver	60	5	150.2	76.2	48.7	68.2
5	56	Liver	45	5	91.2	52.4	31.9	47.8
6	51	Liver	60	5	224.3	65.1	25.0	60.6
7	70	Liver	60	3	26.6	64.7	55.5	61.9
8	64	Liver	60	5	121.5	68.5	26.5	61.5
9	67	Adrenal	50	5	61.0	54.5	45.4	51.6
10	87	Liver	60	5	133.5	75.7	12.1	62.2

### Outcomes

**Figure 1:**
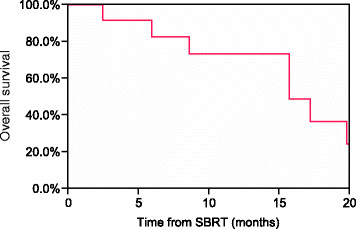
Kaplan-Meier estimate of overall survival for all patients.

**Figure 2:**
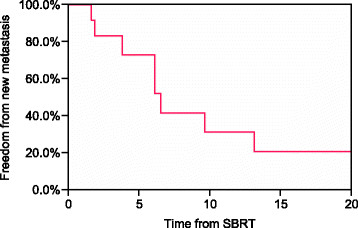
Kaplan-Meier estimate of freedom from new metastasis for all patients.

Clinical outcomes, patterns of failure, and acute and late toxicity for each patient are shown in Table 3. The median follow-up time in living patients was 14 months (range, 2–26 months). At the time of this analysis, 4 patients were dead of disease, 2 patients were dead of other causes, 2 patients were alive with disease, and 2 patients had no evidence of disease. Kaplan-Meier estimates of OS at 6 and 12 months were 83% and 73%, respectively (Figure 1). No patient experienced an in-field failure, but 4 patients treated to liver sites developed a recurrence in a distant portion of the liver. By RECIST, a complete response (CR) was seen in 3 lesions (25%), a partial response (PR) in 5 lesions (42%), and stable disease (SD) in 4 lesions (33%). For those patients experiencing disease progression outside the SBRT field, the median time from SBRT to progression was 6.1 months (range 2–13 months). The 6- and 12-month Kaplan-Meier estimates of FFDM were 73% and 31%, respectively (Figure 2). The most common first sites of disease progression included liver (n = 5), abdominal lymph nodes (n = 4), and lungs (n = 1).

**Table 3:** Outcomes and toxicity

**Patient**	**Site treated**	**Follow-up (months)**	**RECIST score**	**Clinical Outcome**	**FFDM (months)**	**Site of progression**	**Acute toxicity**	**Grade**	**Late toxicity**	**Grade**
1	Liver	25.5	PR	DOD	9.6	Liver (other sites), paracaval LN	Fatigue	1	GI pain, diarrhea	1
	Liver	15.7	PR	DOD	6.1	Paraaortic LN	Nausea	1	GI pain, diarrhea	1
	Lymph node	15.7	PR	DOD	6.1	Paraaortic LN	Nausea	1	GI pain, diarrhea	1
2	Liver	17.2	SD	DOD	13.1	Liver (other sites)	Nausea	2	Biliary stenosis	3
3	Liver	8.6	PR	DOD	1.6	Liver (other sites)	Fatigue	1	GI pain	2
4	Liver	2.4	SD	DOC	-	-	GI pain	1	-	-
5	Liver	21.8	CR	NED	-	-	Nausea	2	-	-
6	Liver	19.8	PR	DOC	-	-	Nausea	2	Liver failure	5
7	Liver	5.9	SD	DOD	3.8	Mesenteric LN	GI pain	2	GI pain	2
8	Liver	11.3	SD	AWD	1.8	Liver (other sites), lung	Nausea, GI pain	2	GI pain	2
9	Adrenal	11.3	CR	AWD	6.5	Liver	-	-	-	-
10	Liver	3.1	CR	NED	-	-	Nausea	2	-	-

Abbreviations: *RECIST* Response Evaluation Criteria in Solid Tumors, *FFDM* Freedom from distant progression, *PR* Partial response, *SD* Stable disease, *CR* Complete response, *DOD* Dead of disease, *DOC* Dead of other causes, *NED* No evidence of disease, *AWD* Alive with disease, *LN* Lymph node, *GI* Gastrointestinal.

### Toxicity

Acute toxicity was common but relatively mild, with no acute Grade ≥3 toxicity reported (Table 3). Common acute toxicities included nausea, fatigue, and abdominal pain. Late toxicity was also common, with all but 4 patients experiencing late side effects. Abdominal pain was the most common late toxicity but was mild in nature. Two patients experienced serious late toxicities; one required placement of biliary stent for Grade 3 biliary stenosis, and another developed Grade 5 liver failure. The patient who developed biliary stenosis did so approximately 6 months after SBRT. He was treated postoperatively to a dose of 50 Gy in 5 fractions for positive resection margins after surgery for intrahepatic cholangiocarcinoma. No dose constraint was employed on the biliary tract, though portions were contained within the high-dose volume. He later developed other sites of metastasis in the liver. The patient who developed Grade 5 liver failure had normal liver function and was Child-Pugh Class A at the time of SBRT and in the immediate follow-up period; however, she had received extensive systemic therapy previously, both for the unresectable cholangiocarcinoma and for metastatic breast cancer 15 years previously. She was treated to a dose of 60 Gy in 5 fractions, and our institutional liver constraint was achieved at the time of treatment planning.

## Discussion

In this small, preliminary report of prospectively-collected data, we demonstrate excellent local control using SBRT for unresectable or recurrent cholangiocarcinoma. Additionally, most patients tolerated treatment well and were able to experience a significant interval free of active disease. A majority of patients in the study eventually progressed distantly or within other hepatic sites; however, one patient is currently alive without evidence of disease nearly 2 years after SBRT, and another patient lived 20 months without evidence of recurrence but died of liver failure. This suggests that SBRT may impact patient survival in addition to local control in appropriately selected patients.

Currently, the most common local treatment approach utilized for unresectable cholangiocarcinoma is fractionated EBRT +/− chemotherapy. A phase II trial of 128 patients with unresectable intrahepatic malignancies treated with concurrent hepatic artery floxuridine and high-dose radiation (median dose 60.75 Gy in 1.5-Gy fractions BID) included 46 patients with cholangiocarcinoma [4]. The median survival for all patients was 15.8 months, with an actuarial 3-year survival of 17%. In that study, 13 of 36 patients (36%) with cholangiocarcinoma who were available for evaluation of treatment response experienced in-field progression, while 15 others progressed in other intrahepatic sites. Another single institution series reported on 52 patients with unresectable extrahepatic cholangiocarcinoma treated with concurrent chemoradiation over a time period of 60 years [5]. Radiation doses and techniques varied widely among the cohort of patients; however, the first site of disease progression was local in 72% of cases, and the median survival was 10 months. Although cholangiocarcinoma is not considered to be curable without surgery, these high rates of in-field progression with standard EBRT +/− chemotherapy imply a more aggressive local treatment approach may be beneficial in properly selected patients.

Newer options showing promise as local therapy for unresectable or recurrent cholangiocarcinoma include TACE, RFA, and PDT [6-8]. A series of 49 patients with unresectable intrahepatic cholangiocarcinoma treated with TACE reported a median survival of 10 months from the time of treatment [7]. The authors found that patients with hypovascular tumors had inferior outcomes compared to those with hypervascular tumors. Another interesting finding was that median survival for tumors <8 cm was 37.2 months and 10.4 months for larger tumors, though this difference was not statistically significant. RFA has shown promise as an effective local therapy, particularly for smaller tumors. For example, a study of 13 patients with 17 primary intrahepatic cholangiocarcinomas treated with RFA reported a crude local control rate of 88% at a median follow-up of 19.5 months. Two local failures occurred, both in tumors >5 cm in diameter. The median overall survival after RFA was 38.5 months. PDT, which involves the interaction of light with photosensitive agents to produce an energy transfer and a local chemical effect, has been tested in a number of small prospective studies with mixed results. A recently-published article summarizes the current medical literature for PDT for unresectable cholangiocarcinoma [8]. Additionally, a small prospective randomized controlled trial of 39 patients comparing PDT after biliary stenting to stenting alone showed a statistically-significant survival benefit favoring PDT [18]. One of the major drawbacks to each of these approaches is that they typically involve an invasive procedure, a problem not encountered with SBRT.

Other groups have reported results using SBRT for cholangiocarcinoma, some of which are summarized in Table 4[12-16]. The largest current series includes 27 patients with unresectable cholangiocarcinoma treated with SBRT [14]. The dose used on all patients in this study was 45 Gy in 3 fractions, prescribed to isocenter. While the authors do not comment specifically on LC, they note median progression-free and overall survivals of 6.7 and 10.6 months, respectively. Interestingly, they found increased rates of duodenal/pyloric ulceration (n = 6, 22%) and duodenal stenosis (n = 3, 11%) compared to other contemporary series, but they were not able to establish a dose-volume relationship for bowel injury. This increased reported rate of bowel injury may be due to the fact that all but one tumor was hilar in location; therefore the high-dose volume was likely located in closer proximity to small bowel. Additionally, the dose and fractionation schema utilized by the Danish group was more aggressive than that of other series. Yet the lack of dose-volume relationship for toxicity implies other underlying and undiscovered factors may also contribute to bowel injury. Other smaller series report one-year LC rates of 65-77% using various dose and fractionation schemes (Table 4). Median survival times vary greatly among these series, likely reflecting patient selection bias. In spite of the smaller patient numbers in the current series, our results compare favorably with those from other institutions, as no patient in our report has experienced a local failure with a median follow-up of more than a year.

**Table 4:** Literature review of SBRT for cholangiocarcinoma

**Reference**	**Year**	**Patients (n)**	**Total Dose (Gy)**	**Fractions**	**12-month LC (%)**	**Median OS (months)**	**Comments**
Herfarth et al. [13]	2001	3	14 - 26	1	71	NR	Target volume covered by 80% isodose
Tse et al. [16]	2008	10	32.5	6	65	15	Hypofractionated stereotactic radiotherapy
Goodman et al. [12]	2010	5	18 - 30	1	77	29	Single-fraction dose escalation study
Kopek et al. [14]	2010	27	45	3	NR	11	22% rate of serious GI injury
Polistina et al. [15]	2011	10	30	3	NR	36	All patients received concurrent gemcitabine
**Current Study**	**2012**	**10**	**55**	**5**	**100**	**14**	**Includes both recurrent and metastatic lesions**

Abbreviations: *LC* Local control, *OS* Overall survival, *NR* Not reported, *GI* Gastrointestinal.

The excellent LC in this series of patients must be balanced against potential toxicity, particularly in light of the Grade 5 liver failure that occurred in one patient. Our institutional liver constraint for 5-fraction liver SBRT, which mirrors constraints provided by the American Association of Physicists in Medicine (AAPM) Task Group 101 (TG101), is to keep ≥700 cm^3^ of normal liver <21 Gy [19,20]. Constraints from the Quantitative Analyses of Normal Tissue Effects in the Clinic (QUANTEC) are slightly more stringent, with recommended mean liver doses of <13 Gy for three-fraction SBRT and <18 Gy for six-fraction SBRT, and ≥700 cm^3^ of normal liver to receive ≤15 Gy [21]. In this patient with a liver volume of 1980.4 cm^3^, 1231.0 cm^3^ of normal liver received <21 Gy and 1051.4 cm3 received <15 Gy, easily meeting both our institutional constraint and the QUANTEC recommendation. The mean liver dose was 19.3 Gy in five fractions, which is slightly higher than the QUANTEC recommendation of 18 Gy for six-fraction SBRT. We have not routinely employed a mean liver dose constraint at our institution when treating with SBRT as the mean dose does not take the size of a patient’s liver into account. Another factor that may have contributed to the patient’s liver failure was her extensive history of previous systemic therapy, which we did not consider during the treatment planning process. This consisted of neoadjuvant doxorubicin, cyclophosphamide, and 5-fluorouracil (5-FU) followed by concurrent 5-FU and postoperative chest wall and nodal irradiation at an outside institution for stage III breast cancer 16 years prior to SBRT. A year later, the patient developed recurrent disease and underwent autologous bone marrow transplantation. This was followed by two years of tamoxifen therapy. The patient was malignancy-free for 13 years, when a new increase in liver function tests led to the work-up and diagnosis of unresectable, intrahepatic cholangiocarcinoma. She received two doses of chemoembolization with mitomycin-C and cisplatin, and then went on to receive nine 28-day cycles of gemcitabine. She had not received any systemic therapy within the previous 12 months of SBRT, nor had she been previously treated with radiotherapy. SBRT was delivered to the previous site of chemoembolization, meaning the previously embolized volume was not considered as part of the ≥700 cm^3^ dose constraint. The patient was Child-Pugh Class A at the time of SBRT though bilirubin, serum albumin, and international normalized ratio (INR) were never checked in the post-SBRT period as she never developed symptoms or side effects of liver failure until she presented at her local emergency department with signs of disseminated intravascular coagulation (DIC). We hypothesize that the patient’s previous systemic therapy and transplant may have resulted in subclinical liver injury, and the additional radiation injury from SBRT depleted what remained of the patient’s already diminished hepatic reserve, enough to cause fulminant liver failure. Indeed, altered liver function is a common complication of stem cell transplantation, with complications ranging from increased liver function enzymes to graft-versus-host disease, hepatic veno-occlusive disease, and death due to liver failure [22]. Interestingly, at the time of liver failure the patient had no evidence of active disease, neither cholangiocarcinoma nor breast cancer.

In summary, this study is a small single-institutional report of patients with unresectable or recurrent cholangiocarcinoma treated with SBRT. With over a year of follow-up, no patient experienced a local recurrence and with one notable exception, the toxicity profile was otherwise acceptable. Although cholangiocarcinoma is a relatively rare malignancy, these results need to be prospectively validated in a larger study. With many other local therapy options available, more information is needed to help physicians stratify patients to the treatments from which they are most likely to benefit. Nonetheless, SBRT appears to be a safe, effective, non-invasive treatment option for carefully selected patients who are unable to undergo surgical resection or who experience an abdominal recurrence of cholangiocarcinoma.

## Competing interests

The authors declare they have no competing interests.

## Authors’ contributions

BB reviewed, analyzed, and interpreted the data, and wrote the manuscript. KO provided significant intellectual contribution and reviewed the manuscript. RM provided significant intellectual contribution and reviewed the manuscript. MH provided significant intellectual contribution and reviewed the manuscript. All authors gave final approval of the manuscript’s submission for publication.
